# Supplementary material for: The effect of prioritization over cognitive-motor interference in people with relapsing-remitting multiple sclerosis and healthy controls
Source: PLoS One. 2019 Dec 23;14(12):e0226775. doi: 10.1371/journal.pone.0226775 (PMC6927625; doi:10.1371/journal.pone.0226775)
Supplement: S1 Table — Abbreviations: pwMS, people with multiple sclerosis; HC, healthy controls; ST, single task; DT-DP, dual task with double priority; DT-CP, dual task with cognitive priority: DTC, dual-task cost; SDMT, Symbol Digit Modalities Test; FDT, Five Digit Test; TAVEC, Test de Aprendizaje Verbal España Complutense; BDI-II, Beck Depression Inventory II; D-FIS, Daily Fatigue Impact Scale; MSQOL, Multiple Sclerosis Quality of Life-54. Note: Values are Spearman’s Rho. D-FIS score is missing from one participant (pwMS n = 22). * p-value < 0.05; ** p-value < 0.001. (DOCX) [file pone.0226775.s002.docx]

**S1 Table. Correlations between CMI parameters and symptomatic features of pwMS and HC**

|  |  | SDMT | FDT- Inhibition | FDT- Flexibility | TAVEC- Immediate Recall | TAVEC- Short-term Recall | TAVEC- Long-term Recall | BDI-II | D-FIS | MSQOL- Global quality of life |
| --- | --- | --- | --- | --- | --- | --- | --- | --- | --- | --- |
| ST distance | PwMS | .587** | -.337 | -.561** | .514* | .458* | .404 | -.250 | -.537* | .535** |
|  | HC | .332 | .065 | .027 | .229 | .042 | .059 | -.409* | -.013 | .369 |
| DT-DP distance | PwMS | .511* | -.277 | -.499* | .472* | .448* | .389 | -.168 | -.519 | .495* |
|  | HC | .357 | -.068 | -.067 | .218 | -.069 | -.101 | -.522** | -.209 | .449* |
| DT-CP distance | PwMS | .295 | -.212 | -.275 | .181 | .125 | .072 | -.123 | -.232 | .432* |
|  | HC | .508* | -.166 | -.355 | -.173 | .032 | .059 | -.486* | -.158 | .217 |
| ST correct words | PwMS | .553** | -.046 | -.308 | .530** | .341 | .439* | -.099 | .190 | .147 |
|  | HC | .399 | -.103 | .518 | .455* | .492* | .606** | -.164 | -.131 | -.018 |
| DT-DP correct words | PwMS | .499* | -.211 | -.429* | .483* | .325 | .451* | -.069 | .009 | .179 |
|  | HC | .614** | -.220 | -.066 | .374 | .253 | .379 | -.393 | -.282 | .049 |
| DT-CP correct words | PwMS | .538** | -.201 | -.556** | .792** | .707** | .742** | .008 | .162 | .072 |
|  | HC | .525** | -.333 | -.036 | .601** | .631** | .778** | -.166 | -.207 | .105 |
| Motor DTC DT-DP | PwMS | .205 | -.256 | -.157 | .164 | .080 | .198 | -.204 | .057 | -.012 |
|  | HC | -.094 | .137 | .147 | .031 | .330 | .303 | .275 | .453* | -.077 |
| Motor DTC DT-CP | PwMS | .323 | -.018 | -.212 | .231 | .261 | .327 | -.100 | -.219 | -.075 |
|  | HC | -.144 | .133 | .344 | .114 | -.008 | .069 | .134 | .130 | .180 |
| Cognitive DTC DT-DP | PwMS | -.151 | .226 | .289 | -.116 | -.108 | -.180 | -.050 | .176 | -.047 |
|  | HC | -.441* | .231 | .161 | -.184 | .090 | -.015 | .400 | .355 | -.196 |
| Cognitive DTC DT-CP | PwMS | -.233 | .181 | .401 | -.426* | -.514* | -.476* | -.101 | .164 | -.025 |
|  | HC | -.135 | .449* | .305 | -.368 | -.357 | -.450* | .089 | .296 | -.148 |

Abbreviations: pwMS, people with multiple sclerosis; HC, healthy controls; ST, single task; DT-DP, dual task with double priority; DT-CP, dual task with cognitive priority: DTC, dual-task cost; SDMT, Symbol Digit Modalities Test; FDT, Five Digit Test; TAVEC, Test de Aprendizaje Verbal España Complutense; BDI-II, Beck Depression Inventory II; D-FIS, Daily Fatigue Impact Scale; MSQOL, Multiple Sclerosis Quality of Life-54. Note: Values are Spearman’s Rho. D-FIS score is missing from one participant (pwMS n=22).

* p-value < 0.05; ** p-value < 0.001
