# Supplementary material for: The effect of prioritization over cognitive-motor interference in people with relapsing-remitting multiple sclerosis and healthy controls
Source: PLoS One. 2019 Dec 23;14(12):e0226775. doi: 10.1371/journal.pone.0226775 (PMC6927625; doi:10.1371/journal.pone.0226775)
Supplement: S2 Table — Abbreviations: pwMS, people with multiple sclerosis; HC, healthy controls; ST, single task; DT-DP, dual task with double priority; DT-CP, dual task with cognitive priority: DTC, dual-task cost. Note: Values are Spearman’s Rho. ERPs data is missing from one participant (pwMS n = 22). * p-value < 0.05; ** p-value < 0.001. (DOCX) [file pone.0226775.s003.docx]

**S2 Table. Correlations between CMI parameters and clinical and physiological features of pwMS**

|  | EDSS | | Disease duration (years) | Number of relapses | Time from last relapse (years) | CzP3 amplitude | CzP3 latency | PzP3 amplitude | PzP3 latency |
| --- | --- | --- | --- | --- | --- | --- | --- | --- | --- |
| ST distance | .658** | | -.006 | -.293 | .126 | .492* | .002 | .499* | -.016 |
| DT-DP distance | -.711** | | -.136 | -.398 | .054 | .447* | -.100 | .467* | -.166 |
| DT-CP distance | -.342 | | .254 | -.056 | .021 | .312 | .142 | .285 | .035 |
| ST correct words | -.058 | | .014 | .245 | -.094 | .086 | .295 | .032 | .260 |
| DT-DP correct words | -.240 | | .145 | .316 | .167 | .370 | .164 | .358 | .084 |
| DT-CP correct words | -.262 | | .071 | .145 | -.224 | .198 | .430* | .145 | .200 |
| Motor DTC DT-DP | .151 | .116 | | .268 | .124 | .111 | .264 | .034 | .239 |
| Motor DTC DT-CP | -.145 | -.199 | | -.059 | .127 | .268 | -.053 | .289 | .049 |
| Cognitive DTC DT-DP | .212 | -.111 | | -.200 | -.205 | -.425* | .036 | -.432* | .053 |
| Cognitive DTC DT-CP | .400 | .036 | | .045 | .176 | -.491* | -.045 | -.467* | .133 |

Abbreviations: pwMS, people with multiple sclerosis; HC, healthy controls; ST, single task; DT-DP, dual task with double priority; DT-CP, dual task with cognitive priority: DTC, dual-task cost. Note: Values are Spearman’s Rho. ERPs data is missing from one participant (pwMS n=22).

* p-value < 0.05; ** p-value < 0.001
